# Barriers to Telemedicine Adoption during the COVID-19 Pandemic in Taiwan: Comparison of Perceived Risks by Socioeconomic Status Correlates

**DOI:** 10.3390/ijerph20043504

**Published:** 2023-02-16

**Authors:** Tzu-Chi Wu, Chien-Ta Bruce Ho

**Affiliations:** 1 Institute of Technology Management, National Chung-Hsing University, Taichung 402, Taiwan; 2 Department of Emergency Medicine, Show Chwan Memorial Hospital, Changhua 500, Taiwan

**Keywords:** socioeconomic status, telemedicine, perceived risk, COVID-19, precision marketing

## Abstract

Telemedicine is the use of technology to deliver healthcare services from a distance. In some countries, telemedicine became popular during the COVID-19 pandemic. Its increasing popularity provides new research opportunities to unveil users’ perceptions toward its adoption and continued use. Existing studies have provided limited information and understanding of Taiwanese users and the various sociodemographic factors that influence their intention to use telemedicine services. Thus, the goals of this study were twofold: identifying the dimensions of perceived risks of telemedicine services in Taiwan and providing specific responses to those perceptions as well as determining strategies to promote telemedicine to local policymakers and influencers by providing a better understanding of the perceived risks in relation to socioeconomic status. We collected 1000 valid responses using an online survey and found performance risk to be the main barrier, which was followed by psychological, physical, and technology risks. Older adults with lower levels of education are less likely to use telemedicine services compared to other categories because of multiple perceived risks, including social and psychological concerns. Understanding the differences in perceived risks of telemedicine services by socioeconomic status may aid in identifying the actions required to overcome barriers and may consequently improve adoption of the technology and user satisfaction.

## 1. Introduction

Telemedicine is a rapidly evolving field that became critical during the COVID-19 pandemic due to social distancing requirements, stay-at-home measures, and travel restrictions [1]. Telemedicine can be an efficient and safe form of providing medical services as it overcomes time and space constraints. The accelerated development of information and communication technologies, including 5G and the Internet of Things, created a favorable environment for the expansion of telemedicine [2]. An increasing number of individuals have benefited from this facility; however, the number of users in countries such as India and Taiwan remain low [3].

During the pandemic, telemedicine was rapidly adopted worldwide, even in countries where it used to be unpopular, such as Taiwan. The crisis enforced the relaxation of restrictions around telemedicine services in Taiwan’s medical law, which originally limited its use to mountainous and other remote areas, outlying islands, or urgent circumstances. Additionally, the new National Health Insurance Law enacted in 2020 now covers telemedicine services [4]. According to current regulations, there are two major ways of utilizing telemedicine: (1) teleconsultations between doctors, especially between different specialists, and (2) patients accessing medical services without the restriction of distance or quarantine [5]. The number of telemedicine beneficiaries has grown exponentially since the pandemic began. However, we currently lack an adequate understanding of the perception of users in Taiwan toward telemedicine service, and most available data are limited to local areas or people still not familiar with the service due to the limits of medical law. A system review about technology acceptance in healthcare points out that Taiwan plays a leading role in the research of technology acceptance in healthcare, but most other studies focus on information systems and electronic medical records solutions [6]. The pandemic has created a new context for telemedicine services in Taiwan, offering new data that differ from the extant research.

Previous studies have stated that unexpected situations and unpredictable events are common reasons that could affect the users’ willingness to adopt certain technologies and result in users’ rejection of technology services. Therefore, numerous studies have explored perceived risks in relation to the societal adoption of such technologies [7]. The perceived risk theory has become popular and has been applied in various fields relating to new product and technology acceptance, such as online shopping behavior [8], Internet banking [9], blockchain [10], and telemedicine [11]. Thus far, studies have evidenced some of the factors that affect the adoption of telemedicine services, including technological availability, lack of knowledge, technology anxiety, perceived risks, time constraints, privacy, and security concerns, but also organizational issues and environmental factors [12]. Most of these studies are varied while sharing only some commonalities.

The pandemic has resulted in the collection of a significant amount of information about the users’ attitudes, behaviors, acceptance level, and the current status of the telemedicine service providers. The use of telemedicine service is not only convenient but also increases patient engagement and adherence to treatment [13]. However, little is known regarding the perceptions about the services of different social categories, especially the vulnerable population including older adults and patients with lower income levels [14]. During the COVID-19 pandemic, the need for technology-based interventions is more pronounced, and it is crucial to design technology-based health solutions for vulnerable populations and older persons, particularly older women [15]. For the most vulnerable populations, the use of telemedicine technology can be difficult to access and benefit from. It is difficult for health providers to break through these barriers. Few studies have focused on the differences in sociodemographic factors which influence the usage intention (UI) of telemedicine among subgroups. Four studies in Taiwan were undertaken in 2015 when the use of telemedicine services was still in its incipient stages; two studies focused on users in Nantou County. To address these research gaps, our study explored the risk perception of telemedicine services among a large population in Taiwan during the COVID-19 pandemic.

To the best of our knowledge, this is the first study on perceived risks about telemedicine in Taiwan examining socioeconomic statuses to explore barriers. An understanding of perceived risks and their socioeconomic correlates can help policymakers and local public health practitioners build a targeted marketing framework and deliver messages to the desired audiences effectively. The results will help in reducing barriers to the adoption of telemedicine services and reduce health-related social disparities among different subgroups.

We define telemedicine service as the provision of medical services and healthcare by suitably qualified providers through the means of information and communication technology without the restriction of physical distance between providers and beneficiaries. Our research objectives were to: (1) identify the dimensions of perceived risks of telemedicine services during the COVID-19 pandemic in Taiwan and (2) investigate specific solutions and promotion strategies to reduce barriers for adoption of telemedicine service.

The rest of the study is organized as follows: Section 2 discusses the theoretical background and formulates the hypotheses; Section 3 details the research methods; and Section 4 presents the results. Section 5 discusses the findings, and Section 6 concludes the study.

## 2. Theoretical Model and Hypotheses

UI is the degree to which “a person has formulated conscious plans to perform or not perform some specified future behavior” [16]. We define UI toward telemedicine services as the intention of a subject to accept remote healthcare services using electronic information technology [17]. Prior studies have also confirmed the direct relationship between the intention to use and the actual use of a service, and many studies use intention as a predictor of the actual use of telemedicine services [11,18]. Therefore, UI is a dependent variable in our study.

According to Bauer’s [19] theory of perceived risk, consumer behavior may produce unexpected results, and a few can be unpleasant. As the outcome of a service and customers’ satisfaction with it can only be evaluated post-acquisition, potential users are compelled to face uncertainty and accept the possible consequences of a mistake—that is, the perceived risk at that time [20,21]. Therefore, the emphasis of the theory is on subjective risk, focusing on the consumers’ subjective expectations of a loss, which varies depending on the culture and organization.

Cunningham [22] introduced six types of risk: financial, performance, psychological, social, time, and safety. Additional dimensions of risk that act as barriers to technology and service adoption, such as privacy, provider-related, physical safety, and technological risks, have been identified in a subsequent stream of literature on online services, including telemedicine [3,23,24,25].

Financial risk (FNR) refers to the potential for monetary loss due to transaction errors or subsequent maintenance cost of the product/service [11]. Uncertainty about insurance coverage and reimbursement for telemedicine service has historically been a major barrier to adoption. Additionally, financial cost is an issue to users, as the cost of installation and maintenance of telemedicine, Internet, and communication equipment is another barrier, especially in developing countries [26]. Performance risk (PFR) is the potential that a product or service will not deliver as much value and desired benefits as required [27]. In a study of 699 mayors in Germany, the highest perceived risk of telemedicine was seen in misdiagnosing and in the concern that the same patient–physician connection would not be established, and thus, the same level of care would not be received compared with face-to-face care [28]. Technology risk (TNR) can be defined as technology failure and the degree to which users perceived uncertainty with telemedicine technology. In both users and healthcare providers, technology risk elicited a negative relationship with UI to adopt telemedicine. Some studies found that technology risk comes from ineffective communication as well as concerns regarding physical limitations and malfunctioning equipment or Internet connection [29].

Perceived psychological risk (PLR) is defined as the consumer’s perception of any possible psychological frustration or anxiety resulting from the use of a service [30]. Prior study considers that use of computer systems can lead to anxiety or emotional reactions, called technology anxiety, which can lead to negative attitudes toward technology adoption [31]. A study among the rural population of Pakistan found that perceived PLR negatively influences UI toward telemedicine services [32]. Moreover, computer anxiety negatively impacts the effort expectancy (related to the degree of ease of use) and affects the use behavior [33]. Social risk (SCR) was defined as a potential loss of status in one’s social group and appearing untrendy because of the service. One worldwide study showed that one of major factors in the overall adoption of telemedicine is peer experience, as telemedicine appears more attractive after watching family and friends use it successfully [34]. In addition to the target group itself, the group’s social environment should be taken into account because potential users can be influenced by others’ opinions and behaviors [35].

Physical risk (PSR) points to the risk of a potential threat to a user’s safety or physical health; in the context of COVID-19, the PSR of telemedicine services relates to the safety and health of the individual minimizing their exposure to COVID-19 [27,36]. Privacy risk (PRR) refers to the possibility of loss of information security of an online user [9]. Privacy concerns are one of the major barriers to online technology adoption because the information utilized includes sensitive personal electronic records and clinical data. This is a factor that negatively impacts UI [37]. Provider risk (PVR) refers to the degree to which users perceive uncertainty with respect to the telemedicine provider [23]. A nationwide survey in the US emphasized the importance of addressing the concern that respondents become less willing to use telemedicine as they become further detached from their own provider [37]. Finally, time risk (TMR) was defined as loss of time and the resulting inconvenience incurred due to delays in finding appropriate services as well as loss of time while learning and using a particular technology [11]. Most users prefer not to use medical services that require significant usage time [38]. To date, most studies point out that telemedicine can reduce physical travel and queue times at the hospital, and the time risk is therefore negligible [34]. Even so, potential users may have concerns about time taken to learn how to use the telemedicine service and may look to replace it with face-to-face consultation if it does not perform to expectations. Research involving 215 doctors in North India showed that social and time risks negatively influenced such usage intentions [11].

**Figure 1:**
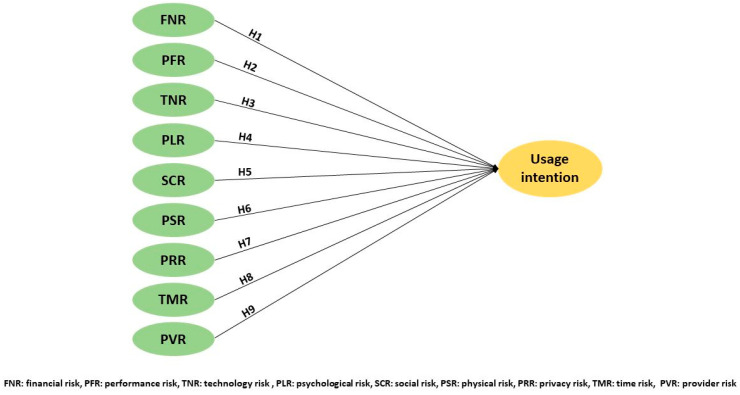
Research model and hypothesis.

Perceived risk is the most often used and reported tool in the assessment of technology acceptance, as it allows the exploration of complex socio-psychological factors [7,11,39]. The results above indicate that the perceived risks of telemedicine differ across countries and roles (user or provider), while the socioeconomic correlates remain unclear. Nine dimensions of risk were selected based on our literature review above, as described in Table 1. We formulated the following research model and nine hypotheses after consideration of these risks (Figure 1).

**H1:** *Financial risk (FNR) negatively influences the UI toward telemedicine services.*

**H2:** *Performance risk (PFR) negatively influences the UI toward telemedicine services.*

**H3:** *Technological risk (TNR) negatively influences the UI toward telemedicine services.*

**H4:** *Psychological risk (PLR) negatively influences the UI toward telemedicine services.*

**H5:** *Social risk (SCR) negatively influences the UI toward telemedicine services.*

**H6:** *Physical risk (PSR) negatively influences the UI toward telemedicine services.*

**H7:** *Privacy risk (PRR) negatively influences the UI toward telemedicine services.*

**H8:** *Provider risk (PVR) negatively influences the UI toward telemedicine services.*

**H9:** *Time risk (TMR) negatively influences the UI toward telemedicine services.*

## 3. Materials and Methods

We conducted a non-interventional questionnaire study and used the social science method to gain an in-depth insight of participants’ perceptions. As the nature of the study was observational, it did not require Institutional Review Board approval of the local Ethics Committee. At the initial stage of distribution of the questionnaire, participants were informed of the purpose of the study and were made aware of the voluntary and anonymous nature of their participation. All procedures followed were in accordance with the Helsinki Declaration.

To understand users’ risk perception of telemedicine services in correlation with different sociodemographic factors, we identified six different groups based on gender, age, education level, monthly income, experience with telemedicine, and history of chronic disease. To improve the quality of the questionnaire, a preliminary version was first tested through an online survey with a pilot study of 50 participants. Three master’s degree holders suggested modifications in the wordings of the items for more conciseness. The responses were rated on a five-point Likert scale, where 1 meant “strongly disagree” and 5 meant “strongly agree.” The cut-off value for Cronbach alpha in all constructs was 0.7, implying the internal consistency and reliability of the summated rating scales [42].

The survey comprised two sections: the first measured the perceived risks and UI toward telemedicine services, while the second captured respondents’ demographic characteristics (Table 2). To ensure the reliability of the results of structural equation modeling, researchers recommend a sample size of minimum 150 and preferably greater than 200 [43]. To analyze subgroups, we collected samples until the size of the smallest group exceeded the lower limit of 150. The online survey of our study was open to the public in Taiwan from 13 June to 30 June 2022. The data were collected anonymously, and a total of 1031 completed questionnaires were received, of which 31 were excluded due to inappropriate answers, and the validity rate was 96.9%. The smallest sample size of a subgroup in the study was 161 cases, and for the other subgroups, it was larger than the recommended guidelines.

The collected data was analyzed using SPSS and SPSS Amos software. Structural Equation Modeling techniques were applied to compare the data and evaluate the measurement model [44], together with Confirmatory Factor Analysis (CFA). Subsequently, SPSS Amos, a statistical software with a graphical interface, was employed for data analysis and hypothesis testing.

### 3.1. Reliability, Validity, and Bias

Five factors—FNR1, TNR1, TNR3, PRR1, and PVR1—were deleted from the CFA to test reliability and validity (Table 3) due to the lower value of factor loadings (the number is the question number). After excluding these, convergent validity and discriminant validity were employed to assess the validity of the model. Convergent validity was significant as the internal consistency reliability for each construct was greater than 0.6 and the Average Variance Extracted (AVE) of all items was greater than 0.50 [45]. The discriminant validity was also considered significant as the AVE was greater than the correlations in that construct’s column or row [46], and the square root of the AVE (diagonal) of the corresponding constructs exceeded the off-diagonal elements in the rows and columns (Table 4) [47]. For common method bias (CMB), variance inflation factor (VIF) was used to evaluate the bias generated by the measurement design and determine how multi-collinear a set of multiple regression variables were with the suggested threshold of 3.3 [48].

### 3.2. Model Fit

**Figure 2:**
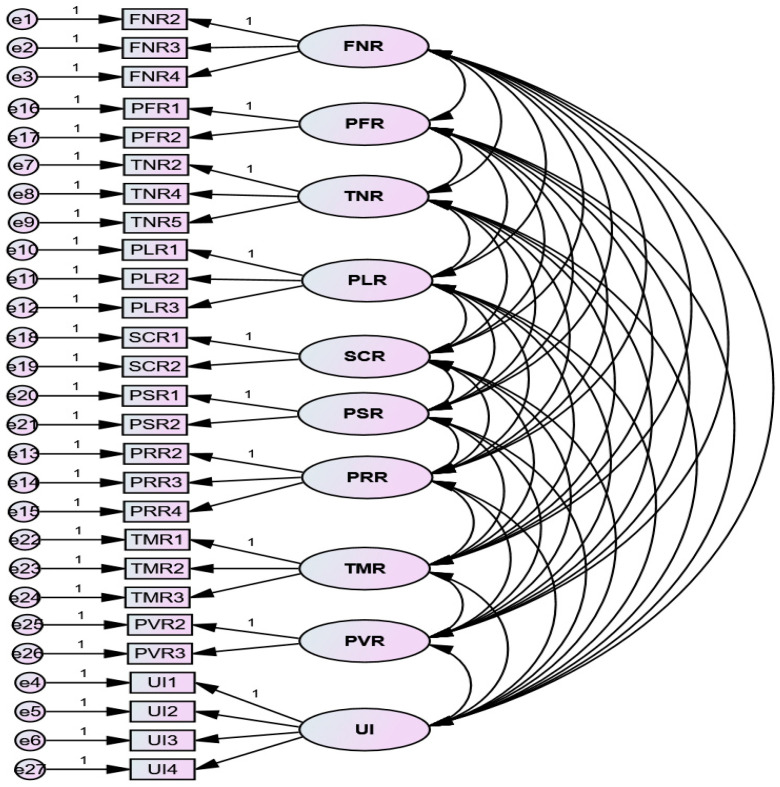
CFA for the measurement model.

The measurement model (Figure 2) was tested using the Chi-square, the ratio of the Chi-square statistics to the respective degrees of freedom (χ^2^/df), the goodness-of-fit index (GFI), the comparative-fit index (CFI) and the root mean square error of approximation (RMSEA) to confirm the fit between the data and the model.

### 3.3. Hypothesis Testing and Group Comparison

**Figure 3:**
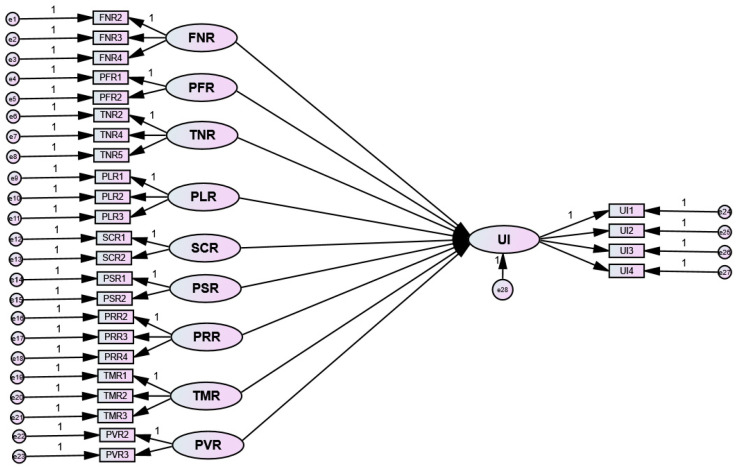
The structural model.

On confirmation of the measurement model, a structural model (Figure 3) was used to explain the relationships among the variables and discover any connection between the constructs, which was followed by hypothesis testing. The bootstrapping resampling technique suggested by Hair et al. [49] examined the model paths, and a *p* value smaller than 0.05 was considered statistically significant.

Finally, six different groups—according to gender, age, education level, monthly income, experience with telemedicine, and history of chronic disease—were individually tested to gain insights in their differences of perceived risks. The older age group is adults aged above 50 years, and the higher-income group is individuals with a monthly income greater than NT$50,000 (>US$1670).

Critical ratios (*z* scores) were used to identify the significant differences between groups on each path of interest. A significant difference was identified for confidence levels of 90%, 95%, and 99%, where the *z* values were 1.65, 1.96, and 2.58, respectively [50].

## 4. Results

The result of the reliability and validity tests in Table 4 indicate adequate convergent validity and discriminant validity. Table 5 provides the details of the model fit. Overall, the measurement model indicated a satisfactory fit. The vales of VIF for all the latent variables in the model are within the range between 1.552 and 2.222, all of which were lower than the suggested threshold, indicating no CMB issues.

Four hypotheses, H2, H3, H4, and H6 (Table 6) were statistically significant. PFR, PLR, TNR, and PSR had a significant negative influence on telemedicine UI, among which PFR was predominant. *Z* scores were calculated to identify the significant differences between subgroups according to gender, age, monthly income, telemedicine experiences, and a history of chronic disease.

**Figure 4:**
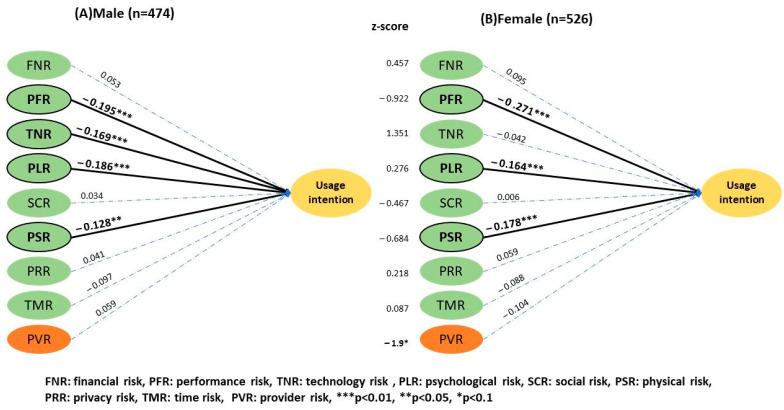
Results of the two-group structural model of gender.

**Figure 5:**
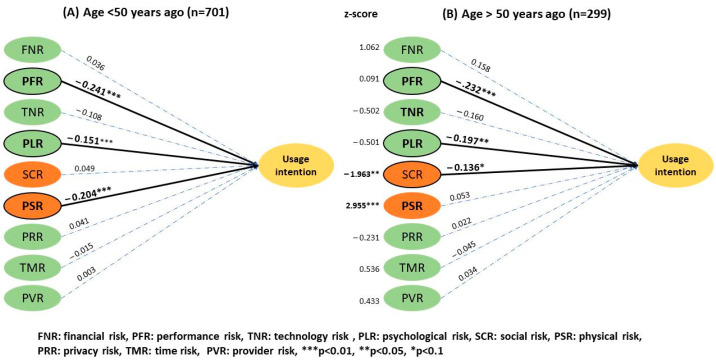
Results of the two-group structural model of age.

Technology risk had a significant effect on telemedicine UI (β = −0.169, *p* < 0.01) among the male population (Figure 4), but it was not significant for women (β = −0.042, *p* > 0.05). There was no significant difference between the two groups with a *z* score of 1.35. The provider-related risk had no significant effect on the UI, but it negatively influenced women’s (3.48 ± 0.92) and positively influenced men’s UI (3.36 ± 1.03), having a *z* score of −1.9 (*p* < 0.1). In relation to age (Figure 5), social risk was (2 ± 0.80) and (2.2 ± 0.78) for the younger and the older group, respectively. Physical risk was (2.4 ± 0.90) and (2.68 ± 0.84) for the younger and the older group, and a significant difference was thus observed between the two groups with *z* scores of −1.963 (*p* < 0.05) and 2.955 (*p* < 0.01), respectively. Users aged above 50 were more concerned with social and physical risk than the younger users.

**Figure 6:**
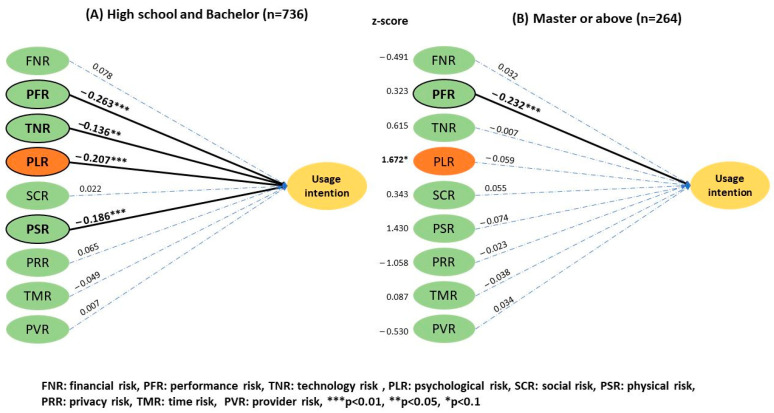
Results of the two-group structural model of educational status.

**Figure 7:**
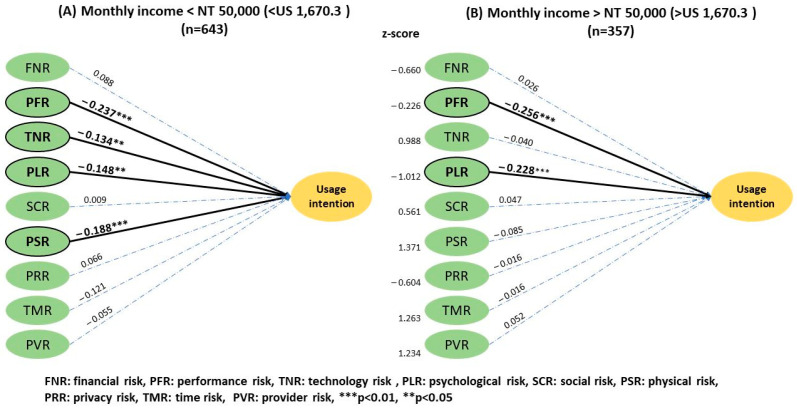
Results of the two-group structural model of monthly income.

The analysis by education level (Figure 6) showed a greater effect on psychological risk in individuals with the highest degree of a high school diploma and bachelor’s (2.75 ± 0.88) than those with a master’s or doctorate degree (2.65 ± 0.92), with a *z* score of 1.672 (*p* < 0.1). The remaining risk factors had no significant effect between the two groups. Additionally, no significant difference was observed in the effect of financial status (Figure 7) on UI between the subgroup with a monthly income lower than NT$50,000 and those with higher incomes.

**Figure 8:**
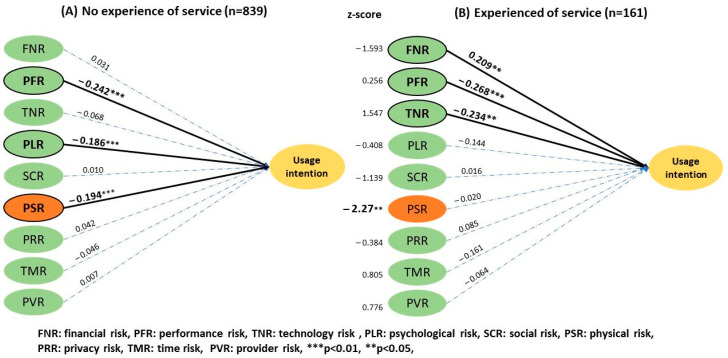
Results of the two-group structural model of experience.

**Figure 9:**
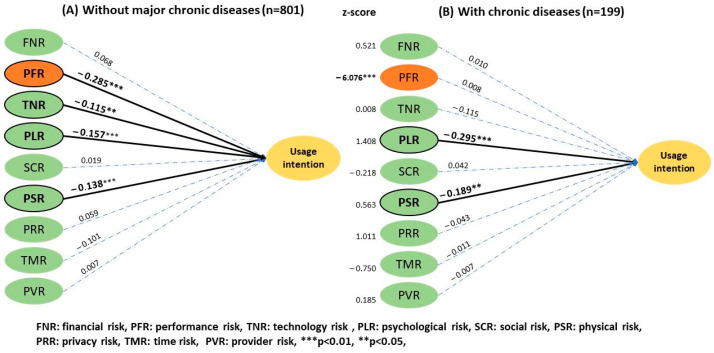
Results of the two-group structural model of health condition.

For telemedicine experience (Figure 8), the subgroup analysis showed that people with no experience (2.53 ± 0.88) perceived a higher physical risk than experienced users (2.26 ± 0.94), which affected their UI. The *z* score was −2.27 (*p* < 0.05). The analysis of the overall health condition (Figure 9) revealed that the group without major chronic diseases perceived a higher performance risk (3.45 ± 0.84) that influenced their UI compared to users with a history of chronic disease (3.17 ± 0.97) with a *z* score of −6.076 (*p* < 0.01).

## 5. Discussion

Communicating and interacting successfully with current and potential beneficiaries of telemedicine represents a crucial task for medical institutions, requiring a good understanding of their typology based on the effective strategies that can be employed to appeal to target markets and generate attention and interest [51]. The COVID-19 pandemic provided a new research context to investigate the resistance of technology adoption and promote the use of telemedicine services in countries where it had not previously been used. The results of the investigations can be practically employed to target various market segments. Our study revealed performance, psychological, physical, and technological risks as common barriers to the adoption of telemedicine services in Taiwan. Recent studies have shown privacy risks are not a barrier toward technology-related healthcare adoption, implying the trust the people of Taiwan have toward information security and the government [32,52]. According to the findings of our study, the need is to overcome the uncertainty of performance, which is the primary solution for enhancing the adoption of telemedicine services by the public.

Previous studies have tried to compare and explain some facets of socioeconomic status differences but with limited evidence. They have shown that people with lower income and education levels, and higher ages, are less inclined to use digital health tools, including telemedicine [53,54]. A systematic review found that older adults and those with lower education level were the least inclined to adopting telemedicine, which correlated with 29% of the responses for lack of UI [55]. Our findings confirm these results and further reveal that the lower education group perceives a higher psychological risk compared to those with higher education. This negatively affects users’ peace of mind, causing nervousness and anxiety, and a fear that remote medical assistance will lead to errors due to lack of experience in technology use.

In relation to age, previous studies have shown that the most significant risk perceived by the older group is caused by technology anxiety, which is related to the lack of computer and Internet experience [31,56]. Fortunately, technology anxiety is not the main barrier among the older group in Taiwan, and this may be due to the high prevalence of smartphone Internet use, with a penetration rate of approximately 90% in 2019. However, this group perceives a higher social risk that stems from the family’s and friends’ perceptions or opinions, affecting the UI. Older adults in Taiwan tend to obtain consent or suggestions from those in their social circle to reduce social risk, worrying about their disapproval [57]. On the contrary, the younger group considers that utilizing telemedicine services decreases their risk of infection, which increases their UI.

In relation to monthly income, an umbrella review emphasized the importance of technology risk in the population with lower income levels since the cost of devices and technological illiteracy may present a barrier for potential users [58]. Interestingly, our analysis showed no significant difference between the higher- and lower-income groups, even though the dividing line was set at a low of <NT$20,000 and <NT$50,000 monthly for the two groups, respectively. Similarly, it has been revealed that the technology risk negatively influences the UI significantly only in the lower monthly income group.

In line with other studies, differences in perceived risks between genders were less salient. Nevertheless, one study found that women were more likely to communicate with health providers through the Internet [59], and another study conducted in the United Arab Emirates revealed that the female group was more likely to use telemedicine services while also reporting higher levels of COVID-19-related fear [60]. Women in Taiwan seem to perceive a higher provider-related risk compared with men, but this does not affect their UI, which may be explained by social role interpretations; for example, women tend to be more risk-averse and prefer visiting their usual doctor and hospital.

Compared to experienced users of telemedicine, those with no experience seem to perceive higher physical and psychological risks, although the latter is not statistically significant. In other words, less fear of physical risk may increase the adoption rate of the service among the inexperienced users. Meanwhile, the experienced group expressed more concerns on performance risks, including the disadvantages of not meeting face-to face or whether the service could adequately solve their medical problems. Similarly, the group without chronic diseases was more concerned with performance and technology risks, which diminished their UI. This perception of risk may stem from less hospital experience, concerns about the lack of physical examination, and the effect of communication with a doctor.

## 6. Conclusions

### 6.1. Theoretical Contributions

Telemedicine is an important innovation and has been an indispensable tool during the COVID-19 pandemic, leading many countries to reassess the status of such services and to optimize their management [61]. Our study provides insights that can correctly guide the promotion of telemedicine services through healthcare providers and policymakers. The latter should recognize and strive to bridge the digital divide caused by social determinants such as age, lack of previous experiences, and health status. To ensure greater adoption and telemedicine equity and sustainability, policy adjustment and precise marketing by service providers should aim to addresses barriers and risks perceived as overwhelming by less advantaged populations [62].

This study evidenced that less advantaged social groups seem to have a higher risk perception hindering their adaptation to the digital healthcare ecosystem. Our results showed that the older users and less educated users are less likely to use telemedicine services as their risk perception is multifaceted compared to their counterparts. To reduce existing disparities and ensure fairness in healthcare provision, clinical and public health communication strategies targeting telemedicine adoption should attend to these differences. The representatives of healthcare and government should engage with stakeholders to redesign and promote virtual care services through efficient and precise marketing, which is essential to ensuring accessibility for all users, especially vulnerable populations.

### 6.2. Practical Implications

Targeted marketing means tailoring promotion strategies to specific audiences to increase the acquisition of products or services. Our study shows that not only the older group but also the less-educated users experience more anxiety and discomfort when considering telemedicine services; increasing their rate of adoption should focus on decreasing their perception of psychological risk. Practical solutions that can be implemented by policymakers include upgrading digital literacy by educating and training patients. Additionally, healthcare providers can employ positive word-of-mouth communication to create the impression of being approachable and friendly. Advertising, training, and even educational training sessions for citizens would help many users to decrease related anxiety and become more comfortable with telemedicine solutions [63].

Furthermore, the group without a history of chronic diseases valued the functionality and effectiveness of the service. Providing more evidence that there is no difference in terms of overall effect and satisfaction between telemedicine services and in-person medical visits and sharing successful experiences may help reduce the doubt about performance [34]. Knowing older users’ preferred information channels, peer groups, living needs, and habits can help select the correct channel to educate and support the patient’s decision making.

### 6.3. Limitations

There are several limitations to our study. First, the methodological choices led to limitations in the research process, as we focused exclusively on the users in Taiwan. As such, the results may not be generalized to other countries. Additionally, all respondents were selected through an online survey, which may have increased the representation of digitally literate individuals in our sample but may produce a bias toward individuals more likely to be online and have Internet access. Furthermore, the study benefited from longitudinal data, but the perceived risk may have to be balanced against time flow, social culture change, and organizational differences. Another limitation concerns UI, which can be mediated by several other factors. Future research may investigate users’ perceived risk in relation to additional sociodemographic predictors, such as ethnicity, religious affiliation, and marital status, allowing policymakers in other countries to adjust their promotion strategy and operations.

## Figures and Tables

**Table 1:** Description of the types of perceived risk.

Type of Perceived Risk	Description	
Financial risk	Risk of monetary loss or unexpected costs due to service and the maintenance costs of the product (such as mobile phone and Internet)	[11]
Performance risk	Risk of not performing as expected or failing to deliver the desired benefits	[40]
Technological risk	Risk of the user’s perceived uncertainty and apprehension (including use, process, and result) regarding the usage of the technology	[23]
Psychological risk	Risk of loss of self-esteem, ego frustration, and generated pressure and anxiety resulting from the service selection	[40]
Social risk	Reflects the disappointment or potential loss of status in the individual by people in surroundings, including friends and family, due to use of the service	[11,20]
Physical risk	Risk to the user’s safety or users fearing risk to their health resulting from the service use	[41]
Privacy risk	Risk of losing personal information and of the stealing of personal health information or data	[40]
Provider risk	Risk of the user’s perceived uncertainty about the service provider, including physicians, nurses, healthcare providers, and hospital	[23]
Time risk	Risk of time lost because of service failure or the service not meeting expectations	[11]

**Table 2:** Sample demographics of 1000 respondents.

Characteristics		Number and Percentage
Gender	Male	474 (47.4%)
	Female	526 (52.6%)
Age	<20	44 (4.4%)
	20–29	274 (27.4%)
	30–39	169 (16.9%)
	40–49	214 (21.4%)
	>50	299 (29.9%)
Education level	High School	129 (12.9%)
	Bachelor’s	607 (60.7%)
	Master’s	241 (24.1%)
	Doctoral	23 (2.3%)
Monthly income in NT$ (US$)	<30,000 (<1002.2)	288 (28.8%)
	30,000–50,000 (1002.2–1670.3)	355 (35.5%)
	50,000–100,000 (1670.3–3340.7)	250 (25%)
	>100,000 (>3340.7)	107 (10.7%)
Residence	Northern Taiwan	344 (34.4%)
	Central Taiwan	432 (43.2%)
	Southern Taiwan	191 (19.1%)
	Eastern Taiwan	33 (3.3%)
Experience with telemedicine	None	839 (83.9%)
	1–3 times	147 (14.7%)
	>3 times	14 (1.4%)
History of chronic disease	None	801 (80.1%)
	Yes	199 (19.9%)

**Table 3:** Reliability, validity test, and means for latent constructs.

Items		Std. Loading	Mean	SD	AVE	CR	* α
Financial risk (FNR)	FNR2	0.72	3.33	0.854	0.585	0.809	0.807
	FNR3	0.77					
	FNR4	0.79					
Performance risk (PFR)	PFR1	0.86	3.40	0.878	0.770	0.870	0.870
	PFR2	0.90					
Technology risk (TNR)	TNR2	0.78	3.45	0.898	0.595	0.815	0.812
	TNR4	0.81					
	TNR5	0.72					
Psychological risk (PLR)	PLR1	0.87	2.73	0.897	0.715	0.882	0.877
	PLR2	0.88					
	PLR3	0.78					
Social Risk (SCR)	SCR1	0.90	2.09	0.811	0.778	0.875	0.874
	SCR2	0.86					
Physical risk (PSR)	PSR1	0.85	2.49	0.897	0.758	0.862	0.859
	PSR2	0.89					
Privacy risk (PRR)	PRR2	0.81	3.42	0.967	0.782	0.915	0.912
	PRR3	0.93					
	PRR4	0.90					
Time risk (TMR)	TMR1	0.54	3.37	0.831	0.506	0.750	0.745
	TMR2	0.75					
	TMR3	0.79					
Provider risk (PVR)	PVR2	0.79	3.43	0.977	0.736	0.847	0.840
	PVR3	0.79					
Usage Intention (UI)	UI1	0.86	3.87	0.768	0.655	0.883	0.882
	UI2	0.82					
	UI3	0.73					
	UI4	0.82					

* α = Cronbach’s α, CR = consistency reliability, SD = standard deviation, AVE = average variance extracted.

**Table 4:** Correlation matrix of variables.

	1	2	3	4	5	6	7	8	9	10
1. FNR	**0.765**									
2. PFR	0.467	**0.878**								
3. TNR	0.532	0.679	**0.772**							
4. PLR	0.459	0.528	0.562	**0.845**						
5. SCR	0.365	0.250	0.327	0.699	**0.882**					
6. PSR	0.392	0.387	0.436	0.563	0.583	**0.871**				
7. PRR	0.562	0.506	0.636	0.463	0.326	0.429	**0.884**			
8. TMR	0.651	0.655	0.668	0.425	0.208	0.314	0.531	**0.712**		
9. PVR	0.596	0.534	0.713	0.483	0.302	0.403	0.763	0.541	**0.858**	
10. UI	−0.217	−0.447	−0.390	−0.399	−0.225	−0.349	−0.248	−0.311	−0.279	**0.809**

FNR: Financial risk, PFR: Performance risk, PLR: Psychological risk, TNR: Technology risk, SCR: Social risk, PSR: Physical risk, PRR: Privacy risk, PVR: Provider risk, UI: Usage intention.

**Table 5:** Fit measures for measurement model.

Fit Measure	Measurement Model	Recommended Values
Chi-square	816	-
Degree of freedom	279	-
χ^2^/df	2.92	<3
GFI	0.941	>0.9
CFI	0.968	>0.9
RMSEA	0.044	<0.08

GFI: Goodness-of-fit index, CFI: comparative-fit index, RMSEA: Root mean square error of approximation.

**Table 6:** Results of hypothesis testing.

Hypothesis (H)		Factor Loading	Standard Error	CR	*p* Valve	Result
H1:	UI<-FNR	0.064	0.045	1.42	0.156	Rejected
H2:	UI<-PFR	−0.242	0.041	−5.96	***	Accepted
H3:	UI<-TNR	−0.116	0.046	−2.53	0.011	Accepted
H4:	UI<-PLR	−0.168	0.039	−4.29	***	Accepted
H5:	UI<-SCR	0.016	0.036	0.44	0.654	Rejected
H6:	UI<-PSR	−0.150	0.036	−4.16	***	Accepted
H7:	UI<-PRR	0.040	0.036	1.11	0.267	Rejected
H8:	UI<-TMR	−0.070	0.055	−1.27	0.203	Rejected
H9:	UI<-PVR	0.005	0.005	0.97	0.331	Rejected

*** *p* < 0.001.

## Data Availability

Not applicable.

## References

[1] Chiesa V., Antony G., Wismar M., Rechel B. (2021). COVID-19 Pandemic: Health Impact of Staying at Home, Social Distancing and “Lockdown” Measures—A Systematic Review of Systematic Reviews. J. Public Health.

[2] West D.M. (2016). How 5G Technology Enables the Health Internet of Things. Brook. Cent. Technol. Innov..

[3] Bakshi S., Tandon U., Mittal A. (2019). Drivers and Barriers of Telemedicine in India: Seeking a New Paradigm. J. Comp. Theor. Nanosci..

[4] Chen T.-H., Ma C.-C., Chiang L.-L., Ou T.-C. (2022). Acceptance of Sustained Utilization Behavior of Telemedicine in the Post-COVID-19 Era. Appl. Ecol. Environ. Res..

[5] Lee P.-C., Wang J.T.-H., Chen T.-Y., Peng C.-H. (2022). Digital Healthcare in Taiwan: Innovations of National Health Insurance.

[6] AlQudah A.A., Al-Emran M., Shaalan K. (2021). Technology Acceptance in Healthcare: A Systematic Review. Appl. Sci..

[7] Gupta N., Fischer A.R.H., Frewer L.J. (2012). Socio-psychological Determinants of Public Acceptance of Technologies: A Review. Public Underst. Sci..

[8] Wu W.-Y., Ke C.-C. (2015). An Online Shopping Behavior Model Integrating Personality Traits, Perceived Risk, and Technology Acceptance. Soc. Behav. Pers. Int. J..

[9] Lee M.-C. (2009). Factors Influencing the Adoption of Internet Banking: An Integration of TAM and TPB With Perceived Risk and Perceived Benefit. Electron. Com. Res. Appl..

[10] Liang P.-H., Chi Y.-P. (2021). Influence of Perceived Risk of Blockchain Art Trading on User Attitude and Behavioral Intention. Sustainability.

[11] Bakshi S., Tandon U. (2022). Understanding Barriers of Telemedicine Adoption: A Study in North India. Syst. Res. Behav. Sci..

[12] Swanson Kazley A., McLeod A.C., Wager K.A. (2012). Telemedicine in an International Context: Definition, Use, and Future. Health Information Technology in the International Context. Adv. Health Care Manag..

[13] Vicente M.A., Fernández C., Guilabert M., Carrillo I., Martín-Delgado J., Mira J.J., Prometeo173 Working Group (2022). Patient Engagement Using Telemedicine in Primary Care During COVID-19 Pandemic: A Trial Study. Int. J. Environ. Res. Public Health.

[14] Eberly L.A., Kallan M.J., Julien H.M., Haynes N., Khatana S.A.M., Nathan A.S., Snider C., Chokshi N.P., Eneanya N.D., Takvorian S.U. (2020). Patient Characteristics Associated With Telemedicine Access for Primary and Specialty Ambulatory Care During the COVID-19 Pandemic. JAMA Netw. Open.

[15] Su Z., Cheshmehzangi A., Bentley B.L., McDonnell D., Šegalo S., Ahmad J., Chen H., Terjesen L.A., Lopez E., Wagers S. (2022). Technology-Based Interventions for Health Challenges Older Women Face Amid COVID-19: A Systematic Review Protocol. Syst. Rev..

[16] Warshaw P.R., Davis F.D. (1985). Disentangling Behavioral Intention and Behavioral Expectation. J. Exp. Soc. Psychol..

[17] World Health Organization (2019). Recommendations on Digital Interventions for Health System Strengthening.

[18] Liu C.F. (2011). Key Factors Influencing the Intention of Telecare Adoption: An Institutional Perspective. Telemed. J. E Health.

[19] Bauer R., Cox D.F. (1960). Consumer Behavior as Risk Taking. Risk Taking & Information Handling in Consumer Behavior.

[20] Maziriri E.T., Chuchu T. (2017). The Conception of Consumer Perceived Risk Towards Online Purchases of Apparel and an Idiosyncratic Scrutiny of Perceived Social Risk: A Review of Literature. Int. Rev. Manag. Mark..

[21] González Mieres C., María Díaz Martín A., Trespalacios Gutiérrez J.A. (2006). Influence of Perceived Risk on Store Brand Proneness. Int. J. Retail Distrib. Manag..

[22] Cunningham S.M., Cox D.F. (1967). The Major Dimensions of Perceived Risk. Risk Taking & Information Handling in Consumer Behavior.

[23] Rho M.J., Yoon K.H., Kim H., Choi I.Y. (2015). Users’ Perception on Telemedicine Service: A Comparative Study of Public Healthcare and Private Healthcare. Multimedia Tool. Appl..

[24] Tandon U., Kiran R., Sah A.N. (2017). Understanding Barriers and Drivers to Online Shopping: An Emerging Economy Case. Int. J. Electron. Bus..

[25] Featherman M.S., Hajli N. (2016). Self-Service Technologies and e-Services Risks in Social Commerce Era. J. Bus. Ethics..

[26] Bajowala S.S., Milosch J., Bansal C. (2020). Telemedicine Pays: Billing and Coding Update. Curr. Allergy Asthma Rep..

[27] Luo X., Li H., Zhang J., Shim J.P. (2010). Examining Multi-dimensional Trust and Multi-faceted Risk in Initial Acceptance of Emerging Technologies: An Empirical Study of Mobile Banking Services. Decis. Support Syst..

[28] Weißenfeld M.M., Goetz K., Steinhäuser J. (2021). Facilitators and Barriers for the Implementation of Telemedicine From a Local Government Point of View-A Cross-Sectional Survey in Germany. BMC Health Serv. Res..

[29] Isautier J.M.J., Copp T., Ayre J., Cvejic E., Meyerowitz-Katz G., Batcup C., Bonner C., Dodd R., Nickel B., Pickles K. (2020). People’s Experiences and Satisfaction With Telehealth During the COVID-19 Pandemic in Australia: Cross-Sectional Survey Study. J. Med. Internet Res..

[30] Yang Y.Q., Liu Y., Li H., Yu B. (2015). Understanding Perceived Risks in Mobile Payment Acceptance. Ind. Manag. Data Syst..

[31] Tsai T.H., Lin W.Y., Chang Y.S., Chang P.C., Lee M.Y. (2020). Technology Anxiety and Resistance to Change Behavioral Study of a Wearable Cardiac Warming System Using an Extended TAM for Older Adults. PLoS ONE.

[32] Kamal S.A., Shafiq M., Kakria P. (2020). Investigating Acceptance of Telemedicine Services Through an Extended Technology Acceptance Model (TAM). Technol. Soc..

[33] AlQudah A.A., Al-Emran M., Daim T.U., Shaalan K. (2022). Toward an Integrated Model for Examining the Factors Affecting the Acceptance of Queue Management Solutions in Healthcare. IEEE Trans. Eng. Manag..

[34] Benis A., Banker M., Pinkasovich D., Kirin M., Yoshai B.E., Benchoam-Ravid R., Ashkenazi S., Seidmann A. (2021). Reasons for Utilizing Telemedicine During and After the COVID-19 Pandemic: An Internet-Based International Study. J. Clin. Med..

[35] Harst L., Lantzsch H., Scheibe M. (2019). Theories Predicting End-User Acceptance of Telemedicine Use: Systematic Review. J. Med. Internet Res..

[36] Portnoy J., Waller M., Elliott T. (2020). Telemedicine in the Era of COVID-19. J. Allergy Clin. Immunol. Pract..

[37] Kato-Lin Y.C., Thelen S.T. (2022). Privacy Concerns and Continued Use Intention of Telemedicine During COVID-19. Telemed. J. E Health.

[38] Topacan U., Basoglu N., Daim T. Health Information Service Adoption: Case of Telemedicine. Proceedings of the 42nd Hawaii International Conference on System Sciences.

[39] Iriani S.S., Andjarwati A.L. (2020). Analysis of Perceived Usefulness, Perceived Ease of Use, and Perceived Risk Toward Online Shopping in the Era of COVID-19 Pandemic. Syst. Rev. Pharm..

[40] Featherman M.S., Pavlou P.A. (2003). Predicting E-Services Adoption: A Perceived Risk Facets Perspective. Int. J. Hum. Comput. Stud..

[41] Carroll M.S., Connaughton D.P., Spengler J.O., Byon K.K. (2014). A Multidimensional Model of Perceived Risk in Spectator Sport. Int. J. Sport Manag. Mark..

[42] Cronbach L.J. (1951). Coefficient Alpha and the Internal Structure of Tests. Psychometrika.

[43] Rigdon E.E., Brian E., David H. (2005). Structural Equation Modeling: Nontraditional Alternatives. Behavioral Science. Encyclopedia of Statistics.

[44] Lowry P.B., Gaskin J. (2014). Partial Least Squares (PLS) Structural Equation Modeling (SEM) for Building and Testing Behavioral Causal Theory: When to Choose It and How to Use It. IEEE Trans. Prof. Commun..

[45] Carlson K.D., Herdman A.O. (2010). Understanding the Impact of Convergent Validity on Research Results. Organ. Res. Methods.

[46] Chin W.W. (1998). The Partial Least Squares Approach for Structural Equation Modeling. Modern Methods for Business Research. Methodol. Bus. Manag..

[47] Fornell C., Larcker D.F. (1981). Structural Equation Models With Unobservable Variables and Measurement Error: Algebra and Statistics. J. Mark. Res..

[48] Kock N. (2015). Common Method Bias in PLS-SEM. Int. JE-Collab..

[49] Hair J.F., Ringle C.M., Sarstedt M. (2011). PLS-SEM: Indeed a Silver Bullet. J. Mark. Theor. Pract..

[50] Hazra A. (2017). Using the Confidence Interval Confidently. J. Thorac. Dis..

[51] Elrod J.K., Fortenberry J.L. (2020). Integrated Marketing Communications: A Strategic Priority in Health and Medicine. BMC Health Serv. Res..

[52] Rad P.M., Chiang D., Connolly J., Mahmood A. (2022). Distance Healthcare in Taiwan. J. Supply Chain Oper. Manag..

[53] Zeng B., Rivadeneira N.A., Wen A., Sarkar U., Khoong E.C. (2022). The Impact of the COVID-19 Pandemic on Internet Use and the Use of Digital Health Tools: Secondary Analysis of the 2020 Health Information National Trends Survey. J. Med. Internet Res..

[54] Nouri S., Khoong E.C., Lyles C.R., Karliner L. (2020). Addressing Equity in Telemedicine for Chronic Disease Management During the COVID-19 Pandemic. NEJM Catal Innov. Care Deliv..

[55] Scott Kruse C.S., Karem P., Shifflett K., Vegi L., Ravi K., Brooks M. (2018). Evaluating Barriers to Adopting Telemedicine Worldwide: A Systematic Review. J. Telemed. Telecare.

[56] Guo X., Sun Y., Wang N., Peng Z., Yan Z. (2013). The Dark Side of Elderly Acceptance of Preventive Mobile Health Services in China. Electron. Markets.

[57] Almousa M. (2011). Perceived Risk in Apparel Online Shopping: A Multi Dimensional Perspective. Can. Soc. Sci..

[58] Eze N.D., Mateus C., Cravo Oliveira Hashiguchi T.C.O. (2020). Telemedicine in the OECD: An Umbrella Review of Clinical and Cost-Effectiveness, Patient Experience and Implementation. PLoS ONE.

[59] Kontos E., Blake K.D., Chou W.Y., Prestin A. (2014). Predictors of E-Health Usage: Insights on the Digital Divide From the Health Information National Trends Survey 2012. J. Med. Internet Res..

[60] Al Meslamani A.Z., Aldulaymi R., El Sharu H., Alwarawrah Z., Ibrahim O.M., Al Mazrouei N. (2022). The patterns and determinants of telemedicine use during the COVID-19 crisis: A nationwide study. J. Am. Pharm. Assoc..

[61] Wu T.C., Ho C.B. (2022). A Narrative Review of Innovative Responses During the COVID-19 Pandemic in 2020. Int. J. Public Health.

[62] Chang J.E., Lai A.Y., Gupta A., Nguyen A.M., Berry C.A., Shelley D.R. (2021). Rapid Transition to Telehealth and the Digital Divide: Implications for Primary Care Access and Equity in a Post-COVID Era. Milbank Q..

[63] Furlepa K., Tenderenda A., Kozłowski R., Marczak M., Wierzba W., Śliwczyński A. (2022). Recommendations for the Development of Telemedicine in Poland Based on the Analysis of Barriers and Selected Telemedicine Solutions. Int. J. Environ. Res. Public Health.

